# Andexanet-Alfa-Associated Heparin Resistance in the Context of Hemorrhagic Stroke

**DOI:** 10.1007/s12028-022-01573-5

**Published:** 2022-08-05

**Authors:** Michael Müther, Wolfram Schwindt, Rolf Michael Mesters, Jens Minnerup, Paul Stracke, Markus Holling, Heinz Wiendl, Walter Stummer

**Affiliations:** 1 Department of Neurosurgery, University Hospital Münster, Albert-Schweitzer-Campus 1, Münster, Germany; 2 Section of Interventional Neuroradiology, Department of Radiology, University Hospital Münster, Münster, Germany; 3 Department of Medicine A-Hematology, Oncology, Hemostaseology, and Pneumology, University Hospital Münster, Münster, Germany; 4 Department of Neurology With Institute of Translational Neurology, University Hospital Münster, Münster, Germany

**Keywords:** Hemorrhagic stroke, Anticoagulants, Heparin, PRT064445, Factor Xa inhibitors

## Abstract

**Background:**

With a growing number of patients on new oral anticoagulants, interest in reversal agents is rising. Andexanet alfa is used for reversal of factor Xa inhibitors in intracranial hemorrhage.

**Methods:**

We provide a brief review on andexanet-alfa-associated heparin resistance and discuss potentially critical situations from different clinical perspectives.

**Results:**

Case reports point out that andexanet alfa can cause unresponsiveness to heparin, leading to catastrophic events. As a result, regulatory bodies have issued warning notices to avoid heparinization parallel to the use of andexanet alfa.

**Conclusions:**

Although well known to hematologists, the phenomenon is underrecognized among stroke clinicians. However, patients with intracranial hemorrhage frequently undergo endovascular or surgical interventions that require periprocedural administration of heparin.

## Background

With a growing number of patients treated with direct oral anticoagulants, general interest in reversal agents in patients with hemorrhagic stroke is rising. Andexanet alfa (AA) is the only reversal agent approved by the European Medicines Agency (EMA) and US Food and Drug Administration for oral direct factor Xa (FXa) inhibitors rivaroxaban and apixaban in situations when reversal of anticoagulation is needed because of life-threatening or uncontrolled bleeding. Safety and efficacy were evaluated in two prospective randomized placebo-controlled studies. Both studies analyzed anti-FXa activity in healthy volunteers receiving either apixaban (ANNEXA-A) or rivaroxaban (ANNEXA-R) [1]. This was followed by the ANNEXA-4 study published in 2019 on 352 patients who had acute major bleeding within 18 h after administration of an FXa inhibitor [2]. Just recently, the post hoc ANNEXA-4 substudy on hemostatic efficacy and anti-FXa reversal in intracranial hemorrhage (ICH) was published [3]. The authors conclude that AA reduced anti-FXa activity in FXa-inhibitor-treated patients with traumatic as well as spontaneous ICH with high rates of hemostatic efficacy. Results of the ongoing ANNEXA-I trial (NCT03661528), a randomized controlled trial evaluating the efficacy and safety of AA versus usual care in patients with ICH on FXa inhibitors, are excitedly awaited. In the absence of such a randomized controlled trial, safety and efficacy are being questioned by different groups of health care professionals, especially when considering the high cost of therapy with AA [4, 5].

## AA-Associated Heparin Resistance

**Fig. 1:**
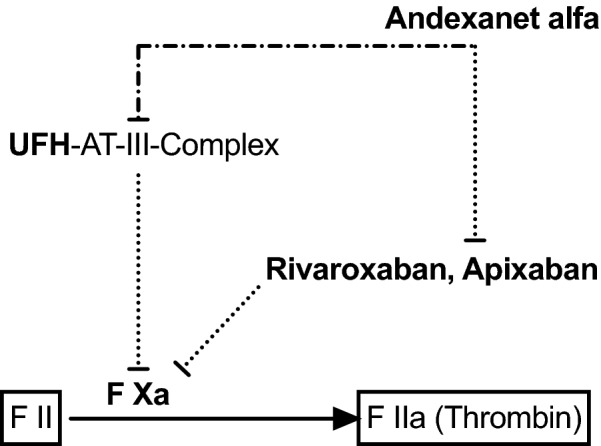
Simplified schematic outline of andexanet-alfa-associated heparin resistance. Rivaroxaban and apixaban inhibit factor Xa (FXa), which normally catalyzes the formation of thrombin (dotted line). Andexanet alfa reverses the effects of apixaban and rivaroxaban by binding with higher affinity to these molecules compared with endogenous FXa (dotted line). Unfractionated heparin (UFH) potentiates the antithrombin (AT)-mediated inhibition of FXa. Andexanet alfa also binds to heparin-activated AT and thereby neutralizes the effect of heparin (intersected line)

AA is a recombinant modified FXa decoy protein that has been shown to reverse the effects of direct FXa inhibitors apixaban and rivaroxaban by binding with higher affinity to these molecules compared with endogenous FXa. Although it was developed as an antagonist for direct FXa inhibitors, AA has also been shown to bind to antithrombin-dependent FXa inhibitors, such as unfractionated heparin (UFH), via binding to heparin-activated antithrombin [6, 7] (Fig. 1). These findings even led to the conclusion that AA may be suitable as an alternative to protamine in reversal of UFH [8].

UFH is administered during open vascular and endovascular procedures to reduce the risk of thromboembolic events. The use of AA prior to interventions with intended heparin anticoagulation has been reported to cause unresponsiveness to heparin. Few case reports from the field of cardiovascular surgery were published pointing out this problem [9–11]. All publications report clinical cases of patients who underwent reversal of direct FXa inhibitors with AA for life-threatening hemorrhage caused by either aortic aneurysm rupture or pericardial tamponade and subsequently developed critical intraoperative thromboembolism in the setting of periprocedural heparin administration (Table 1). Despite the supratherapeutic dose of UFH, point-of-care testing using activated clotting time failed to demonstrate a therapeutic heparinization that was necessary for cardiopulmonary bypass circulation [9–11]. Intraoperative UFH resistance is a well-known phenomenon in the field and is often attributed to antithrombin deficiency, increased UFH clearance, elevation in UFH binding protein, or high levels of factor VIII or fibrinogen [12, 13]. Administration of AA prior to heparinization was identified as another potential cause of heparin resistance. As a result, AA is now critically discussed in the cardiothoracic surgery community [14–16]. The above-mentioned case reports, together with further suspected adverse events reported to the EMA, have led to publication of a direct health care professional communication that was disseminated in November 2020. Although extent and duration of the interaction have not been evaluated, the EMA recommends avoiding the use of AA before heparinization [17].

**Table 1:** Published case reports of andexanet-alfa-associated heparin resistance

Author	Anticoagulant (indication)	Underlying condition	Andexanet alfa dose regimen	Indication for heparinization (dose)	Signs of thromboembolism	Action taken (outcome)
Apostel et al. [9]	Apixaban (atrial fibrillation)	Pericardial tamponade after elective radiofrequency ablation for atrium flutter	Low dose regimen	Open drainage of pericardial tamponade under cardiopulmonary bypass (80,000 IU UFH)	Clots in surgical field and bypass circuit	Discontinuation of andexanet alfa, 1000 IU antithrombin (no permanent morbidity)
Eche et al. [10] and Watson et al. [11]	Rivaroxaban (atrial fibrillation)	Ruptured abdominal aortic aneurysm	Low dose regimen	Emergency endovascular aneurysm repair (14,000 IU UFH)	Thrombus occlusion of left internal iliac artery	Discontinuation of andexanet alfa and application of fresh frozen plasma (no permanent morbidity)

The second case listed was published twice

IU, international units; UFH, unfractionated heparin

Once identified as such, standardized protocols to address AA-associated heparin resistance are lacking. One single case of successful antithrombin substitution can be appreciated [9]. Another group reports on administration of fresh frozen plasma [11]. Both strategies were adopted from management of heparin resistance in coronary bypass surgery, in which antithrombin deficiency was found to be a major risk factor [18]. Because AA has been shown to bind to heparin-activated antithrombin, raising the overall concentration of AA is a potential salvage strategy [18]. For fresh frozen plasma, the effect on heparin resistance is highly speculative [19]. If the application of AA was followed by heparinization, we recommend to immediately stopping the AA infusion protocol. Because data supporting indications of clinical benefit with antithrombin supplementation outside of cardiac surgery are lacking, we propose treatment with direct thrombin inhibitors argatroban or bivalirudin [20]. These agents are favorable because of their therapeutic effect downstream of AA in the coagulation cascade [11]. Importantly, all these substances have not been studied in detail for treatment of AA-associated heparin resistance.

## Special Considerations in the Management of Complex Hemorrhagic Stroke

Despite doubts about the evidence for use of AA among patients with hemorrhagic stroke, the substance is currently being used in clinical practice [5]. In the management of secondary-cause ICH (e.g., aneurysmal subarachnoid hemorrhage, ruptured arteriovenous fistulas, or arteriovenous malformation), microsurgical open vascular or endovascular procedures may be necessary to timely address the source of bleeding. Importantly, such procedures may well require temporary heparinization. Because the cardiothoracic community has already embraced the notion that AA should not be used for patients requiring heparinization, this issue has not yet been discussed within the neurovascular community [16]. With no data on AA-associated heparin resistance in patients with stroke available, the potential hazard of this association mandates translation of findings from cardiovascular medicine into stroke care. The distinct vascular physiology of cerebral circulation makes thromboembolism even more likely to cause harm here than in the extremity vasculature, which occurred in two cardiovascular case reports. Within the following paragraphs we sought to emphasize potential caveats of AA-associated heparin resistance and the need for vigilant management among all different angles of care to help prevent dramatic and potentially life-threatening situations.

## Acute Stroke Care Perspective

**Fig. 2:**
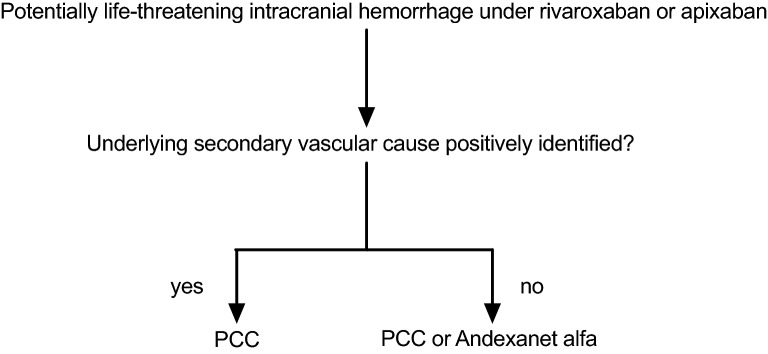
Institutional decision management algorithm for patients with potentially life-threatening intracranial hemorrhage under rivaroxaban or apixaban. PCC, prothrombin complex concentrate

For primary care facilities, AA administration can be a valuable part of the initial resuscitative bundle prior to transfer of a patient to a stroke referral center. AA would normalize coagulation parameters during the highest risk period of interfacility transport, and its short half-life would allow it to clear by the time the patient arrives at the referral center [11]. Importantly, AA reverses FXa inhibitor levels for only 3 h, and then levels return to baseline, as compared with treatment with placebo. The pharmacokinetic half-life, however, is 5–7 h. As a result, heparin resistance may last for several hours after infusion [1, 2]. Conversely, a safe starting time for heparin has not been established. Starting AA before transport to a referral center can therefore be associated with potential morbidity for the patient. Ideally, the decision to administer AA should be made collaboratively between different specialties involved in hemorrhagic stroke care. To ease this decision-making process, we have established a simple administration algorithm at our institution (Fig. 2). On the basis of available computed tomography or magnetic resonance vascular imaging, each case is reviewed by an interdisciplinary team consisting of an attending neurologist, a neurointerventionalist, and a neurosurgeon. If a secondary vascular cause, such as a ruptured aneurysm or other vascular malformation (with potential need for heparinization during further treatment), is identified, a decision will be taken to administer a prothrombin complex concentrate at a dose of 50 IU/kg body weight instead of AA to address coagulopathy associated with direct oral anticoagulants. This is a situation in which AA should certainly not be administered. Importantly, considering limited evidence on safety and efficacy, a clear recommendation to administer AA over prothrombin complex concentrate in all other situations cannot me made. Including a pharmacist in the interdisciplinary team evaluating the optimal anticoagulation reversal strategy based on patient characteristics may help with decision-making. If AA was started without interdisciplinary review, the neuroendovascular and surgical teams will be notified, and decision-making processes can be adapted accordingly.

## Surgical Perspective

The AA approval study does not provide any safety or efficacy data that can be extrapolated to the setting of a surgical procedure because the study excluded surgical patients. Still, optimal and timely hemostasis is highly desirable prior to any open intracranial neurosurgery. Several groups have already reported on the successful use of AA prior to hematoma evacuation [21–23]. However, AA-associated heparin resistance can be a problem when complex open neurovascular procedures, such as bypass surgery for trapping of a complex ruptured aneurysm, require heparinization to avoid formation of thromboembolism at the suture sites [24].

## Neurointerventional Perspective

Patients with intracerebral hemorrhage frequently undergo acute endovascular interventions for treatments of aneurysms, arteriovenous malformations, or fistulas. Importantly, thromboembolic events represent the most frequent procedure-related complication [25]. Underlying causes include prothrombotic effects of endothelial injury, thrombus formation on the catheter hardware, and reduced flow in access route vessels and their vascular territories [25, 26]. Although practices of coagulation management vary, it is widely accepted that heparinization should be used to prevent thromboembolic complications during and after the procedure [27–30]. Notably, heparin resistance during neurovascular interventions may not be encountered as frequently as in many vascular surgery settings, where cardiopulmonary bypass and higher doses of UFH are often necessary. Here, heparin resistance can occur in up to 22% [18]. Although during endovascular procedures, repetitive angiography allows for early recognition of thromboembolic events, and thrombectomy via the same route can be executed immediately, repetitive vascular manipulation in the setting of AA-associated heparin resistance may be ineffective and can lead to a higher degree of local thrombosis and thromboembolism.

## Conclusions

Stroke clinicians need to understand that AA can lead to heparin resistance with serious thromboembolic complications. It is crucial to early identify patients potentially undergoing procedures that necessitate periinterventional heparinization. To our knowledge, this is the first appreciation of AA-associated heparin resistance in acute neurological care literature, and we invite colleagues to discuss and apply our institutional decision support algorithm. More evidence is needed to appropriately judge the clinical significance of AA-associated heparin resistance in acute neurovascular diseases.
